# Facial expression recognition reveals students’ engagement in online class: Correlations with six engagement measurements

**DOI:** 10.1371/journal.pone.0334232

**Published:** 2025-10-22

**Authors:** Xuhui Hu, Jian Gao

**Affiliations:** 1 School of International Education, Shantou University, Shantou, China; 2 School of Chinese as a Second Language, Peking University, Beijing, China; University of Lahore - Raiwind Road Campus: The University of Lahore, PAKISTAN

## Abstract

Student engagement assessment in online learning faces critical limitations: traditional methods fail to capture engagement’s dynamic, multidimensional nature, particularly in second language (L2) contexts where emotional factors are paramount. This study introduces a novel multi-method framework that combines real-time facial expression recognition with dynamic self-reporting and observational measures to provide comprehensive, temporally-sensitive engagement assessment in synchronous online classrooms. Through analyzing a focused sample of Chinese L2 learners, we examined correlations between automatically detected happiness expressions and six established engagement measurements across both static and dynamic scales. Results revealed a significant and robust correlation between happiness expressions and self-reported emotional engagement, representing the study’s primary validated finding. Additional correlations were found with specific mood indicators (happy and loving items). However, no significant correlations were observed with behavioral engagement, cognitive engagement, or flow experience. The facial expression recognition successfully captured dynamic engagement fluctuations, showing modest but significant correlations with both retrospective self-reports and classroom observations. Our findings demonstrate that facial expressions serve as valuable indicators of specific engagement dimensions in online L2 learning, offering a targeted tool for emotional engagement assessment within comprehensive, multi-method evaluation frameworks in digital education.

## Introduction

Student engagement is widely recognized as a crucial determinant of educational outcomes, particularly in language learning contexts [1]. However, previous research has encountered two significant challenges: a lack of conceptual clarity in defining engagement and methodological limitations, particularly the predominant reliance on single-time self-report measures, including self-reported engagement, mood assessments, flow measurements, and classroom observations [2–5]. These traditional methods often fail to capture the dynamic nature of engagement and may be subject to various forms of response bias [6]. These limitations are particularly pronounced in online learning environments, where traditional indicators like body language may not be directly applicable due to limited visibility of non-verbal cues and technical barriers [7,8]. As online learning continues to grow, the inability to accurately measure student engagement risks misinterpreting performance, undermining instructional quality, and impeding the development of effective teaching strategies [9].

To address these challenges, researchers have begun exploring more dynamic and objective measures of engagement. Decades of psychological research, including recent studies [10–13], have established strong correlations between facial expressions and emotional states, supporting the classic classification in the field of psychology [14]. This body of work suggests that facial expressions can provide valuable insights into individuals’ affective experiences, which are closely linked to engagement [2,15]. Concurrently, advancements in computer-assisted facial expression recognition technology have emerged as a promising tool for objective and continuous assessment [16,17].

While facial expression recognition for engagement assessment has been explored in educational contexts, existing research has predominantly focused on generic learning environments and relied on single-method approaches that fail to capture engagement’s multidimensional complexity. Guided by action research principles [18], we introduce a novel integrated framework that combines real-time facial expression analysis with dynamic self-reporting and observational measures, specifically designed and validated for second language (L2) learning contexts.

## Literature review

### The evolution of student engagement in L2 learning

The concept of engagement in language learning, initially introduced by Dörnyei and Kormos [19], has become central to understanding successful second language acquisition [20,21]. Despite its acknowledged importance, fewer than 35% of studies offer clear operational definitions of engagement [2], leading to considerable variations in research scope and outcomes. This definitional ambiguity emphasizes the urgent need to establish clear conceptual frameworks in engagement research.

The theoretical understanding of engagement has evolved substantially over time. Early conceptualizations viewed engagement as students’ full psychological and physical investment in learning activities [1]. Building on this, Fredricks et al. refined this conceptualization by defining engagement as a multidimensional construct encompassing behavioral, emotional, and cognitive dimensions [22], which has been widely adopted in subsequent research in L2 learning.

Each dimension of engagement contributes uniquely to the learning process. Behavioral engagement manifests through active participation in learning activities, serving as an observable indicator of student involvement [23]. Emotional engagement encompasses students’ affective reactions to the learning environment, forming the foundation for other engagement types [24]. Cognitive engagement involves students’ psychological investment in mastering complex concepts and has been identified as a crucial predictor of academic achievement [25].

Recent research has expanded this framework to include social engagement, examining the quality and frequency of learner interactions during instructional activities [26]. Studies have demonstrated positive correlations between social engagement and learning outcomes [27], with evidence suggesting that collaborative tasks enhance motivation and performance in L2 settings [26]. Furthermore, Ben-Eliyahu et al.‘s investigation into the relationships between behavioral, emotional, and cognitive engagement has deepened our understanding of how these dimensions interact and influence overall engagement [28].

While the multidimensional framework has been extensively applied in traditional classrooms, the rapid transition to online learning environments has introduced new considerations for engagement research [29]. Synchronous online teaching, which facilitates real-time interactions akin to face-to-face settings [30], faces unique challenges such as limited non-verbal communication and technical barriers, which can heighten student anxiety and disrupt engagement [7,31,32]. For example, Hu indicated variations in question allocation measures and waiting times between online and offline teaching [33]. Similarly, Ji et al. identified that technical issues heightened student anxiety, resulting in reduced emotional engagement and satisfaction among learners [8], highlighting how online-specific factors complicate the application of traditional engagement models.

Despite its conceptual diversity, an emerging consensus defines engagement as a multidimensional construct [2,34]. This study adopts Fredricks et al.‘s framework [22], for its robust delineation of behavioral, emotional, and cognitive dimensions, which are particularly suited to analyzing task-level engagement in synchronous online L2 teaching, as described by Hiver et al. as operating on a timescale of minutes or hours [2].

### Measurement approaches in student engagement research

Current methodologies for measuring student engagement in language learning employ diverse approaches across behavioral, emotional, and cognitive dimensions. Behavioral engagement assessment typically focuses on quantifiable metrics such as word counts [19], time and frequency invested in learning endeavors [35,36], and the degree of learners’ active involvement in the learning process [37]. These measurements primarily rely on statistical analyses derived from classroom observations [38,39] and survey responses from both students and instructors [5].

The measurement of emotional and cognitive dimensions presents greater complexity due to their less observable nature. Researchers have traditionally relied on student self-reporting to assess these aspects. Emotional engagement has been evaluated through various frameworks, including discrete emotion scales measuring enjoyment, anxiety, and boredom [23,40], as well as independent mood scales [41,42] and flow measurements [43]. Flow, representing an optimal state of intrinsic motivation where learners become fully immersed in their activities, has emerged as a particularly valuable indicator of engagement [44]. Additionally, researchers have explored non-verbal indicators of emotional engagement, such as instances of laughter during classroom interactions [45], and the presence of smiles as markers of active emotional involvement [46,47].

Cognitive engagement assessment has primarily focused on students’ self-reported attention levels [48], as well as their self-regulation and learning strategies used [49,50]. Some studies have expanded their scope to include verbal manifestations of cognitive engagement, examining peer interactions, student questioning patterns [51], and language-related episodes involving content elaboration and clarification [26].

However, current measurement methodologies face two significant limitations. First, there is an overreliance on subjective self-reporting methods, with surveys and questionnaires accounting for 37.5% of measurements and interviews or focus groups comprising 30.3% [2]. These methods are inherently susceptible to cognitive bias and social desirability [6]. Second, most traditional approaches fail to capture the dynamic nature of engagement, providing only static snapshots rather than continuous measurement.

In response to these limitations, researchers have explored alternative approaches. One direction involves neurophysiological measurements to capture both cognitive states and emotional responses [52]. These studies have utilized specialized equipment to monitor eye movements, heart rate variations, and EEG patterns [53–55]. While promising, these methods often require sophisticated laboratory settings, limiting their practical application in typical educational contexts.

Another emerging approach involves continuous reporting methodologies, including both concurrent and retrospective reporting techniques [56]. Concurrent reporting requires participants to document their emotional states at frequent intervals during the learning [57], while retrospective reporting involves participants reviewing and reporting their emotional states after completing learning tasks [58].

Various tools have been developed to facilitate these real-time assessments, such as the Affective Slider for rapid emotion evaluation [59], and mobile applications for tracking student motivation and engagement across different time periods [60]. Notable innovations include Classmoto, a program developed specifically for online courses that allows teachers to assess multiple dimensions of engagement through real-time class-wide queries [61]. These developments acknowledge and respond to the dynamic nature of engagement within individual lessons.

Objective observational methods have emerged as a valuable complement to self-report measures, offering reduced bias in engagement assessment [37]. Researchers have refined these approaches by segmenting teaching sessions into short intervals for detailed behavioral and emotional classification [5]. The field has increasingly moved toward integrated methodological frameworks. Skinner et al.‘s influential work demonstrated the value of combining behavioral and emotional engagement measurements, revealing significant correlations between observational data and survey responses [5,62]. Building on this foundation, Bae et al. developed a comprehensive approach that successfully integrates quantitative surveys with qualitative classroom observations [63], providing a more complete picture of student engagement.

### Facial expression recognition and research questions

Recent advances in facial expression recognition technology have opened new possibilities for objective and continuous assessment of student engagement [64,65]. The development of automated facial expression recognition systems, particularly those utilizing deep learning and hybrid neural networks, has enabled researchers to analyze students’ emotional states and engagement levels with unprecedented precision and temporal resolution [66,67].

These advances have enabled several pioneering educational studies. Ninaus et al. employed Microsoft’s Emotion API alongside traditional emotion scales to demonstrate higher emotional involvement in game-based learning compared to conventional approaches [17]. Their research validated the potential of facial expression recognition as a complementary tool for engagement assessment. Similarly, Tonguç and Ozkara extended this methodology to analyze dynamic changes in student emotions during traditional lectures [68], providing valuable insights into temporal patterns of engagement.

Goldberg et al. advanced engagement research by integrating manual coding with machine learning to predict student engagement [69]. They combined indicators like gaze direction, head positioning, and facial expressions, finding strong correlations with observer ratings in face-to-face settings. Their work also suggested that peer interactions influence engagement, underscoring the role of social dynamics in classrooms. In a parallel line of inquiry, Alkabbany et al. investigated behavioral and emotional engagement by leveraging multimodal features such as facial fiducial points, head pose, and eye gaze [15]. Subsequent work further refined academic affective states into discrete categories, including boredom, confusion, focus, frustration, yawning, and drowsiness, thereby enhancing the granularity of engagement analysis [70]. Building on this foundation, they further introduced a happiness dimension to the framework, aligning emotional taxonomies more closely with classroom-specific contexts and diversifying engagement assessment metrics [71]. Collectively, these studies underscore the evolving sophistication of facial expression analytics in capturing nuanced engagement dynamics within educational settings.

In online learning contexts, facial expression recognition has gained traction as a tool to overcome the limitations of traditional engagement indicators. Zhang et al. developed an end-to-end network for automatic student engagement recognition, achieving high accuracy in binary classification on the DAISEE dataset [10]. Similarly, Siswantoro et al. (2024) applied a convolutional neural network (CNN) to detect engagement in online lectures, preprocessing video frames (e.g., grayscale conversion, face detection) to classify students as engaged or disengaged [13]. Complementing this, Savchenko et al. introduced a privacy-preserving neural network that jointly classifies emotions and engagement [12].

Notably, recent research efforts have begun integrating facial analytics with self-reported psychological measures. Buono et al. developed an LSTM-based multimodal framework analyzing facial action units, gaze patterns, and head movements in relation to self-reported engagement. Their analysis showed a relatively weak overall correlation between facial behavior and emotional engagement, with more pronounced connections for positive affect. Specifically, positive emotional engagement demonstrated stronger alignment with action units activation (ρ = 0.37) while exhibiting an inverse relationship with gaze behavior (ρ = −0.36) [11]. The self-report instrument in this study operationalized engagement through adapted gamified learning scale [72] and the User Engagement Scale short-form [73].

The convergent evidence from facial expression research aligns with theoretical expectations about the manifestation of positive emotional engagement [5]. According to component process models of emotion [74], emotional experiences involve coordinated responses across multiple systems, including facial expressions as observable indicators of underlying affective states. When students experience positive emotional engagement should manifest in detectable facial expressions, particularly those involving smile-related muscle activity [75].The moderate effect sizes observed in previous research (r = 0.37–0.45) are theoretically meaningful and practically significant [11,69]. They suggest that facial expressions capture a substantial portion of positive emotional engagement while acknowledging that engagement is a complex, multifaceted construct that cannot be fully assessed through any single indicator. This partial overlap between facial expressions and engagement measures supports a multi-method approach to engagement assessment, where facial expressions provide complementary information to traditional self-report and observational measures.

Despite these advances, current facial expression recognition research in education faces three critical limitations that our study addresses. First, existing studies have predominantly focused on generic learning environments [76,77], overlooking domain-specific contexts like L2 acquisition where emotional engagement plays uniquely critical roles in learning outcomes [78,79]. Furthermore, cultural differences significantly influence how emotions are expressed and interpreted through facial cues [80,81], posing challenges for applying generic facial recognition models in L2 settings. Second, most research has employed single-method approaches, limiting the ability to comprehensively validate facial expression analysis against multiple established engagement measures within unified frameworks. Third, existing studies have typically examined either static or dynamic patterns, but not both, missing crucial insights that emerge from temporal dual-perspective analysis. This study addresses these gaps by introducing a multi-method framework specifically designed for L2 contexts that integrates facial expression recognition with six established engagement measures across both temporal scales [22,34]. This approach enables us to determine not only whether facial expressions correlate with traditional measures, but also what unique insights facial analysis provides that other methods cannot capture.

Previous research has indicated that while neutral expressions predominate in educational settings [68], happiness expressions occur with notable frequency and may serve as reliable indicators of active engagement [46,47]. This observation informs our focus on analyzing happiness expressions as potential markers of student engagement. Specifically, our research addresses two key questions:

1. From a static perspective, what correlations exist between mean happiness expression levels and various self-reported measures of engagement, including behavioral, emotional, and cognitive engagement, flow experience, and mood changes?
2. From a dynamic perspective, how do happiness expressions fluctuate during class sessions, and how do these variations correlate with real-time retrospective self-reports and classroom observations?

## Method

This study was approved by the Ethics Committee of the School of International Education at Shantou University (approval number: 50121001) and was conducted in accordance with the ethical standards outlined in the 1964 Declaration of Helsinki and its later amendments. Participants were recruited through multiple channels, including online advertisements, institutional invitations, and verbal announcements by course instructors during their classes. All participants provided informed consent electronically before their inclusion in the study. Participants were fully informed about the purpose, procedures, and their rights, including the right to withdraw at any time. Data collection and analysis were conducted in strict adherence to principles of confidentiality and anonymity.

### Participants and context

This exploratory study adopted an intensive sampling approach to facilitate an in-depth analysis of engagement patterns in synchronous online learning environments. This method was selected to prioritize detailed data collection from a smaller, purposively chosen group over broad generalizability, aligning with the study’s aim to explore correlations between happiness expressions and engagement measures. Initially, one hundred and two from 28 countries were recruited, all using English as the primary language of instruction. Inclusion criteria were: (1) age 18 or older, (2) prior experience in synchronous online classes, (3) current Chinese L2 learners, and (4) ability to complete English questionnaires.

Participant selection also adhered to specific criteria related to camera use and survey completion, reflecting the typical conditions of synchronous online learning environments. While camera use was encouraged at the study’s outset, it was not mandated, consistent with common practices in synchronous online teaching. Following the application of inclusion criteria, 46 participants were excluded (42 due to insufficient camera use during sessions, and 4 due to incomplete survey responses), resulting in a final sample suitable for facial expression analysis. The final analysis included data from 60 participants (17 beginners and 43 intermediate-level learners) for facial expression and real-time measurement analysis, and 54 participants (10 beginners and 44 intermediate-level learners) for single-time measurement analysis. This selective sampling enabled intensive individual-level analysis and detailed examination of engagement patterns, providing rich data for this novel methodology.

The participant pool comprised two distinct proficiency levels: beginners with one semester of Chinese language learning experience and intermediate learners with more than one year of study. To ensure instructional consistency while maintaining ecological validity, the course structure followed a standardized format across all sessions, including lead-in activities, vocabulary instruction, text comprehension, grammar practice, and cultural components. Two experienced instructors, each with approximately five years of teaching Chinese as a second language, conducted the classes.

### Measurements

To evaluate student engagement across behavioral, emotional, and cognitive dimensions, the study employed six validated measurement instruments, selected for their established reliability in educational research and compatibility with facial expression recognition. These included: the Brief Mood Introspection Scale (BMIS) to assess emotional states; the Short Flow Scale (SFC) to measure cognitive absorption; the Engagement vs. Disaffection with Learning (EvsD) scale to evaluate behavioral and emotional engagement; the Motivated Strategies for Learning Questionnaire (MSLQ) to gauge cognitive engagement through self-regulation; automated facial expression recognition to capture real-time happiness expressions; and retrospective self-reports paired with classroom observations to assess dynamic engagement. Each instrument was chosen to provide a multidimensional perspective on engagement, complementing the study’s innovative use of facial recognition technology. A detailed description of these instruments, including their psychometric properties, is provided in Table 1.

**Table 1:** The summary of measurement in this study.

Measures	Dimensions of Test Engagement		
	Behavioural	Emotional	Cognitive
NON-REAL TIME MEASUREMENTS			
The Brief Mood Introspection Scale (BMIS) (Mayer & Cavallaro, 2019)		√	
Short Flow Scale(SFC) (Jackson et al., 2008)		√	
Education versus Disaffection with learning (EvsD) (Skinner et al., 2009)	√	√	
Motivated Strategies for Learning Questionnaire (MSLQ) (Pintrich & Groot, 1990)			√
REAL TIME MEASUREMENTS			
Retrospective Students’ Reports based on Affective Slider (Betella & Verschure, 2016)		√	
Observation (Skinner et al., 2009)	√		
Facial Expression Recognition		√	

**The Brief Mood Introspection Scale (BMIS)** was implemented to assess participants’ emotional states through 16 distinct mood items including Lively, Happy, Sad, Tired, Caring, Content, Gloomy, Jittery, Drowsy, Grouchy, Peppy, Nervous, Calm, Loving, Fed up, and Active. Participants indicated their current mood state using a 4-point Likert scale ranging from “definitely do not feel” (1) to “definitely feel” (4).

**The Short Flow Scale (SFC)** evaluated nine distinct dimensions of flow experience: Challenge Skill Balance, Action Awareness Merging, Clear Goals, Unambiguous Feedback, Concentration on Task at Hand, Sense of Control, Loss of Self-consciousness, Transformation of Time, and Autotelic Experience. This scale provided insights into participants’ immersive learning experiences.

**The Engagement versus Disaffection with Learning (EvsD)** scale offered a multidimensional assessment framework encompassing both behavioral and emotional aspects of engagement and their corresponding disaffection indicators. The behavioral component evaluated students’ effort, attention, and persistence through five items focused on learning activity participation. The emotional component assessed both positive involvement and indicators of withdrawal or alienation during learning activities through 17 items. Two school-context questions were removed from both behavioral dimensions to maintain classroom-specific relevance: “I try hard to do well in school” and “I don’t try very hard at school.”

**The Motivated Strategies for Learning Questionnaire (MSLQ)** provided measures of cognitive engagement through selected dimensions of self-regulation and learning strategy use. To maintain a manageable survey scope, we focused on two representative dimensions commonly employed in L2 cognitive engagement research, excluding items not directly related to classroom cognitive engagement [49,50]. Two items unrelated to current classroom cognitive engagement were excluded: “When I do homework, I try to remember what the teacher said in class so I can answer the questions correctly” and “I work on practice exercises and answer end-of-chapter questions even when I don’t have to.”

**Facial Expression Recognition** methodology captured participants’ expressions throughout the learning sessions through camera recordings at 10-second intervals [68]. These images were processed using Microsoft Emotion Recognition API, which generates intensity scores (0–1) for eight basic emotions: anger, contempt, disgust, fear, happiness, neutral, sadness, and surprise. This data enabled the creation of temporal emotion line chart throughout the learning sessions.

**Retrospective Students’ Reports** utilized the Affective Slider to assess real-time emotional engagement during online learning sessions [59]. During synchronous classes, researchers recorded teaching content and students’ video feeds. Students then reviewed the recordings and reported their emotional states at 2-minute intervals using the Affective Slider’s Pleasure and Arousal dimensions. This retrospective approach was selected based on research validating its correlation with concurrent reporting methods, while minimizing classroom disruption [53,82].

Observation followed Skinner et al.‘s coding framework, adapted for online learning environments [5]. The modified scheme categorized behaviors into four types: On-Task Active (Reading, Writing notes, Asking or answering academic questions); On-Task Passive (Listening to the teacher or a classmate making an on-task active contribution); Off-Task Active (Disrupting a classmate or interrupting the teacher with a non-academic issue); Off-Task Passive (The learner is facing the camera, but the portrait is not visible in the video, or the learner’s eyes leave the screen for an extended period). Two trained coders analyzed classroom videos at 15-second intervals, generating approximately 8 codes per participant per minute to maintain consistency with established standards [5].

### Data analysis

The analysis of facial expression data required careful consideration of its relationship with other engagement dimensions. Initial examination of the data distribution using the Shapiro-Wilk test revealed non-normal distributions for both individual happiness expression values and overall mean expressions (p < 0.01). Based on these findings, we selected Spearman rank correlation analysis as the most appropriate method for examining relationships between facial expression data and other measurement dimensions.

To address our two primary research questions, we analyzed facial expression data from both static and dynamic perspectives. For the static analysis, we computed the mean happiness expression value for each participant across the entire teaching session and examined its correlations with single-time self-report measures, including: a)The positive-negative emotion scores and the difference of individual mood items from the BMIS, b)Mean flow scores from the difference of SFC, c)Mean behavioral and emotional engagement scores, including their sub-dimensions from the EvsD, d)Mean cognitive engagement scores and their sub-dimensions from the MSLQ.

To ensure consistent measurement across different instruments, standardized preprocessing steps were applied. In line with Skinner et al. [5], negatively worded items in the EvsD scale, such as those assessing behavioral and emotional disaffection (e.g., ‘I don’t try very hard at school’), were reverse-coded prior to calculating mean scores. Concurrently, in adherence to the specifications for the MSLQ [83], designated items marked with (R) indicators (e.g., “When work is hard I either give up or study only the easy parts”) were reflected before scale construction. These standardization procedures minimized potential bias from mixed item wording directions, ensuring valid comparisons in subsequent correlation analyses.

For the dynamic analysis, we established a standardized two-minute time interval as the basic unit of measurement across different assessment methods. This allowed us to compute the mean happiness expression values within each interval and analyze their correlations with pleasure and arousal scores from the retrospective self-reports. We conducted Kappa consistency tests to assess inter-coder reliability for classroom observations before analyzing correlations between happiness expressions and both primary (On-task/Off-task) and secondary behavioral categories.

### Factor analysis

A two-stage factor analytic procedure was implemented to provide rigorous psychometric validation: exploratory factor analysis (EFA) to investigate the underlying factor structure, followed by Bayesian confirmatory factor analysis (CFA) to test the hypothesized measurement model. Prior to confirmatory analysis, EFA examined the factor structure of happiness expressions and emotional engagement measures. Data suitability was assessed using the Kaiser-Meyer-Olkin (KMO) test and Bartlett’s test of sphericity. Factor number determination employed both Kaiser’s eigenvalue-greater-than-one criterion and parallel analysis [84,85]. A single-factor EFA was performed using maximum likelihood estimation without rotation, appropriate for the two-variable analysis. Bayesian Confirmatory Factor Analysis (CFA). Based on EFA results, a Bayesian CFA was conducted using the bcfa function in R’s blavaan package to test a single-factor model with happiness expressions and self-reported emotional engagement as indicators of a latent “positive emotional engagement” construct, based on the correlations between the two as studied by previous researchers [5,11,69].

Bayesian priors were informed by previous research and theoretical considerations. For self-reported emotional engagement, a prior of normal (0.7, 0.15) was specified based on Skinner et al., who found that student-reported emotional engagement loadings typically ranged from 0.55–0.84 [5]. For facial happiness expressions, a prior of normal (0.4, 0.1) was set based on previous reported correlations of r = 0.45 between facial expressions and engagement in face-to-face settings [69], and correlations of ρ = 0.37 in online learning contexts [11]. The analysis employed 4 chains with 10,000 burn-in iterations and 20,000 sampling iterations to ensure convergence. Model fit was evaluated using posterior predictive p-values (PPP), with values around 0.5 indicating good fit. Convergence was assessed using Rhat statistics, with values ≤ 1.1 indicating successful convergence.

### Procedure

The study implementation followed a carefully structured protocol to ensure data quality and participant comfort. Upon entering the virtual meeting room, participants received comprehensive information about the study’s objectives and procedures, followed by obtaining informed consent. The protocol emphasized participants’ autonomy, including their right to withdraw from the study at any point and to exclude their data from analysis if desired.

To establish a standardized emotional baseline, all consenting participants viewed a seven-minute documentary collectively. This preliminary step aimed to mitigate potential pre-existing emotional influences that might affect the subsequent measurements [86,87]. Following this standardization phase, participants completed their first BMIS and SFC assessment before proceeding to the 35-minute teaching session.

The post-teaching phase involved a systematic sequence of assessments. Participants first completed the second BMIS and SFC, followed by the EvsD and MSLQ scales through electronic questionnaires. After a brief intermission, researchers introduced the retrospective self-reporting methodology and provided detailed instructions for using the Affective Slider. To ensure accurate data collection, participants engaged in practice sessions with the Affective Slider, though data from these practice rounds were explicitly excluded from the statistical analysis.

The formal reporting phase employed a structured approach to data collection. Researchers or their assistants shared screens displaying the recorded class video. The playback process incorporated regular two-minute intervals, at which points the video was temporarily paused. During these pauses, participants received verbal prompts to report their emotional states using the Affective Slider, specifically focusing on their experiences during that particular timeframe. This cycle of video playback and emotional state reporting continued systematically until the conclusion of the recorded session.

## Result

### Results from single-time measurement

**Fig 1:**
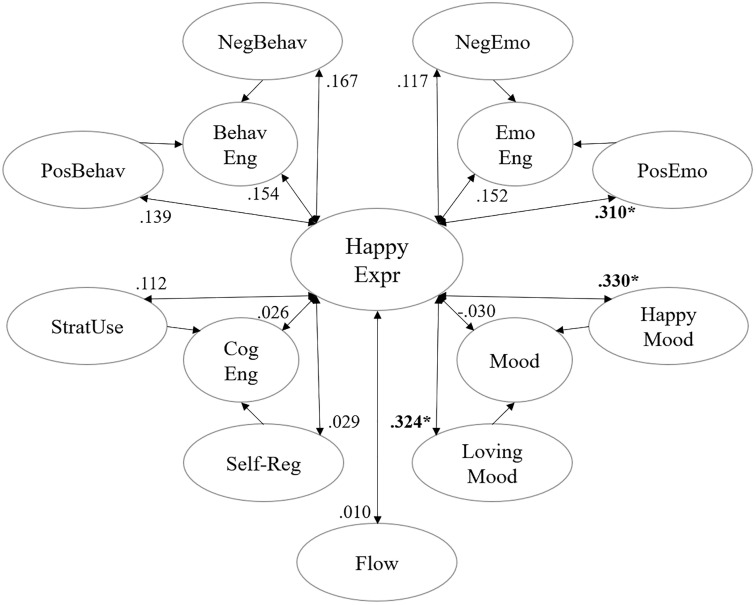
Correlation between happiness expression and single-time engagement measurement result.

The analysis demonstrated significant positive correlations between happiness expressions and key engagement metrics. Descriptive statistics for these variables are presented in Table 2, with comprehensive correlation matrices available in S1 Appendix. Fig 1 visually summarizes these relationships, illustrating their strength and direction.

**Table 2:** Descriptive statistics of happiness expression and single-time measurements.

	N	M	SD	Min (Max)	Skewness	Kurtosis	Reliability (α)
HappyExprAvg	54	0.0287	0.0438	0.00 (0.15)	1.676	1.378	/
BEHAVIOURAL ENGAGEMENT (EVSD)							
BehavAvg	54	4.3194	0.6237	2.50 (5.00)	−0.898	0.502	0.888
BehavPos	54	4.4815	0.5723	2.75 (5.00)	−1.17	1.05	
BehavNeg	54	4.1574	0.8072	1.00 (5.00)	−1.378	3.266	
EMOTIONAL ENGAGEMENT (EVSD)							
EmoAvg	54	4.3878	0.5228	2.71 (5.00)	−1.208	1.75	0.951
EmoPos	54	4.4889	0.6052	2.80 (5.00)	−1.029	0.321	
EmoNeg	54	4.3457	0.5272	2.67 (5.00)	−1.102	1.567	
COGNITIVE ENGAGEMENT (MSLQ)							
CogAvg	54	3.694	0.4528	1.63 (4.68)	−1.957	7.509	0.832
StratUse	54	4.003	0.5695	1.45 (4.73)	−1.921	6.349	
SelfReg	54	3.2685	0.4131	1.88 (4.75)	−0.562	6.037	
MOOD (BMIS)							
MoodScorded	54	54.9074	7.0128	30.00 (64.00)	−1.897	4.17	Before:0.835; After:0.916
HappyMood	54	0.4444	1.0218	−2.00 (3.00)	−0.12	0.271	
Flow(SFC)	54	0.2176	0.366	−0.87 (1.13)	0.331	1.803	Before:0.876; After:0.92

The EvsD measurements demonstrated high internal consistency (α = 0.951), with strong reliability in both behavioral (α = 0.888) and emotional (α = 0.951) sub-dimensions. These dimensions showed significant intercorrelations (p < 0.01), supporting the instrument’s construct validity. Happiness expressions correlated significantly with positive emotional engagement (ρ = 0.310, p = 0.022) but showed no significant associations with behavioral engagement, behavioral disaffection or emotional disaffection (p > 0.1). Further analysis revealed non-significant correlations with behavioral engagement (ρ = 0.154, p = 0.265), reverse-coded behavioral disaffection (ρ = 0.167, p = 0.227), and overall emotional engagement (ρ = 0.152, p = 0.274). Emotional disaffection also showed no significant correlation (ρ = 0.117, p = 0.398).

The MSLQ analysis demonstrated high reliability for cognitive strategy use (α = 0.832). However, we found no significant correlations between cognitive engagement and other engagement dimensions (p > 0.1), nor between happiness expressions and any cognitive engagement measures (p > 0.1).

The BMIS showed strong internal consistency both before (α = 0.835) and after (α = 0.916) the teaching session. Analysis of emotional changes during the teaching process revealed an interesting pattern: while the mean happiness expressions showed no significant correlation with the overall positive-negative scale (ρ = –0.030, p = 0.828). However, a significant positive correlation was observed with the item “happy” (ρ = 0.330, p = 0.015), and the item “loving” (ρ = 0.324, p = 0.017). No other mood items showed significant associations (all p > 0.05). Detailed correlations between happiness expressions and all 16 BMIS mood items can be found in S2 Appendix.

The SFC demonstrated robust internal consistency across both pre-class (α = 0.876) and post-class (α = 0.920) measurements. However, correlation analysis revealed no significant relationship between happiness expressions and flow experience (ρ = 0.010, p = 0.941). S3 Appendix provide the detail results of single-time measurement.

### Results of factor analysis

Data suitability tests confirmed appropriateness for factor analysis (KMO = 0.50, marginally acceptable; Bartlett’s test: χ² = 5.97, df = 1, p = 0.015). Both Kaiser’s criterion (eigenvalue = 1.331 > 1) and parallel analysis recommended a single-factor solution. The single-factor EFA revealed equal loadings for both variables (λ = 0.575), with the factor explaining 33.08% of the total variance. Communalities were identical for both indicators (h² = 0.331), suggesting equivalent contributions to the underlying construct. The equal factor loadings indicate that facial expressions and self-reported emotions contribute equally to the measurement of positive emotional engagement, providing evidence for measurement equivalence across different assessment methods.

The Bayesian CFA demonstrated acceptable model fit (PPP = 0.414) with excellent convergence (all Rhat ≤ 1.001). Standardized factor loadings were: EmoPos (λ = 0.805, serving as the reference indicator) and HappyExprAvg (λ = 0.572, 95% CI: [0.012, 0.128] unstandardized). The CFA results provided preliminary evidence for convergent validity, with both indicators loading significantly on the positive emotional engagement factor.

Construct validity assessment revealed Average Variance Extracted (AVE = 0.459) and Composite Reliability (CR = 0.629). While the AVE falls below the conventional 0.50 threshold [88], this may reflect the theoretical complexity of emotional engagement rather than measurement inadequacy. Component process models of emotion [74] suggest that emotional experiences involve multiple, partially independent response systems—including facial expressions, subjective feelings, and behavioral tendencies—that while correlated, capture distinct aspects of the emotional experience. The modest AVE therefore indicates that facial expressions and self-reported emotional engagement represent complementary rather than redundant indicators of emotional engagement.

This theoretical interpretation is supported by several converging lines of evidence: (1) convergent validity from specific mood correlations, where happiness expressions correlated significantly with self-reported “happy” (ρ = 0.330, p = 0.015) and “loving” moods (ρ = 0.324, p = 0.017), providing additional triangulation; (2) all standardized factor loadings exceed 0.57 and are statistically significant; (3) composite reliability (CR = 0.629) meets acceptable thresholds for exploratory research; and (4) factor loading consistency between EFA (0.575) and CFA (0.572) approaches.

However, we acknowledge important limitations in our measurement model, including the modest AVE value and relatively small sample size, which suggest that our findings should be interpreted with caution and require validation with broader samples and more robust psychometric evaluation in future research (Complete measurement statistics are provided in S5 Appendix).

### Results from real-time measurement

**Fig 2:**
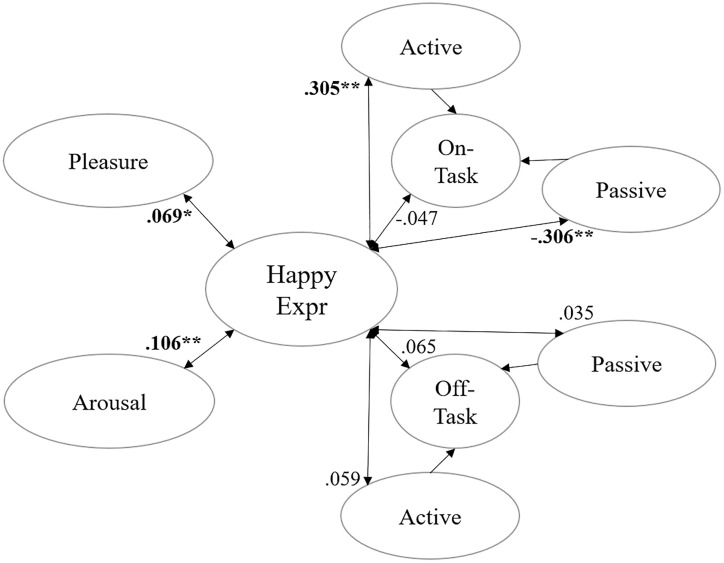
Correlation between happiness expression and real-time engagement measurement results.

The real-time measurement analysis revealed dynamic patterns in student engagement throughout the learning sessions. Table 3 presents the descriptive statistics for facial expression recognition and real-time engagement measurements, with detailed correlation analyses provided in S4 Appendix. Fig 2 illustrates the relationships between happiness expressions and real-time measurement variables.

**Table 3:** Descriptive statistics of happiness expression and real-time measurements.

	N	M	SD	Min (Max)	Skewness	Kurtosis
HappyExpr	877	0.0289	0.0811	0.00 (0.82)	4.552	27.49
CLASSROOM OBSERVATION						
On-Task	877	0.9962	0.0272	0.63 (1.00)	−8.653	83.714
On-Act	877	0.0637	0.1077	0.00 (0.81)	2.031	5.443
On-Pas	877	0.9325	0.1097	0.19 (1.00)	−1.92	4.815
Off-Task	877	0.0031	0.0257	0.00 (0.38)	9.57	101.472
Off-Act	877	0.0009	0.0105	0.00 (0.13)	11.499	131.897
Off-Pas	877	0.0022	0.0235	0.00 (0.38)	11.611	142.444
STUDENT SELF-REPORTS						
Pleasure	877	0.7755	0.139	0.31 (1.00)	−0.54	−0.364
Arousal	877	0.8161	0.1406	0.44 (1.00)	−0.746	−0.247

**Fig 3:**
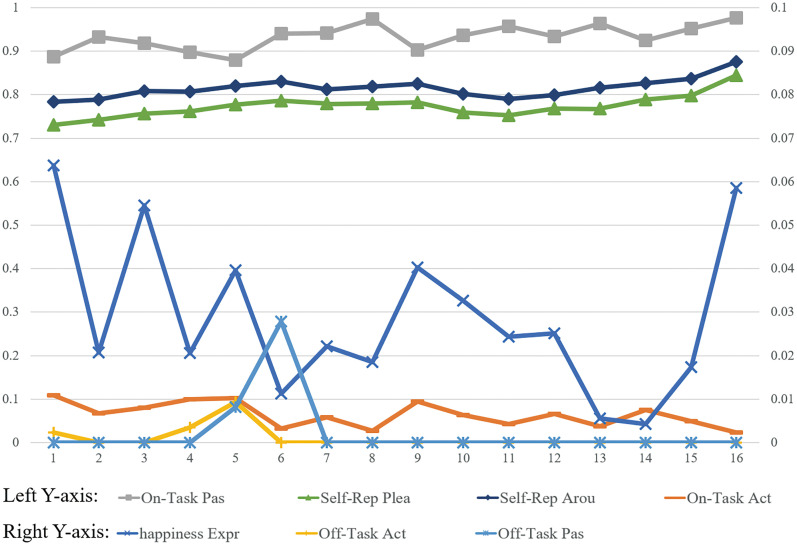
Contrasting Real-Time Results of Happiness Expression, Classroom Observation, and Student Self-Reports.

To facilitate comparative analysis, we standardized all real-time engagement measurements on a 0–1 scale, using two-minute intervals as the basic unit of analysis. The results revealed distinct patterns in engagement indicators: observed on-task passive behavior and retrospective self-reported measures (both pleasure and arousal) showed consistently high proportions, exceeding 0.75. In contrast, happiness expressions, observed on-task active behavior, and both categories of off-task behavior demonstrated notably lower proportions, all falling below 0.11. Fig 3 illustrates these temporal patterns using separate y-axis coordinates to accommodate the different scales of measurement.

The analysis of happiness expressions revealed distinct temporal patterns throughout the teaching sessions. Expression levels typically began high at session commencement, followed by a decline, then showed a notable increase during the middle portion of the session, particularly during grammar and practice activities. After another brief decline, expression levels rose rapidly toward the session’s conclusion, reaching their peak in the final minutes.

Classroom observation analysis, based on every 15 seconds coded segments, demonstrated high inter-rater reliability (Kappa κ = 0.931). The distribution of observed behaviors showed clear patterns: on-task passive behavior dominated (M = 0.933, SD = 0.110), followed by on-task active behavior (M = 0.064, SD = 0.108) and off-task passive behavior (M = 0.002, SD = 0.024), with off-task active behavior occurring least frequently (M < 0.001, SD = 0.011).

Happiness expressions showed statistically significant correlations with several observation-based engagement indicators. Specifically, they were positively correlated with on-task active behavior (ρ = 0.305, p < 0.001) and negatively correlated with on-task passive behavior (ρ = –0.306, p < 0.001). Correlations with off-task indicators were weak and non-significant (off-task active: ρ = 0.059, p = 0.081; off-task passive: ρ = 0.035, p = 0.300), and no significant correlation was found with the overall on-task proportion (ρ = –0.047, p = 0.165). These results are summarized in Table S2 (S4 Appendix).

The retrospective self-reports revealed consistently high levels of both pleasure (M = 0.776, SD = 0.139) and arousal (M = 0.816, SD = 0.141), with a strong positive correlation between these two dimensions (ρ = 0.882, p < 0.001). Analysis of individual participants’ self-reports showed that happiness expressions were significantly but modestly correlated with both pleasure (ρ = 0.069, p = 0.042) and arousal (ρ = 0.106, p = 0.002), supporting a weak but consistent link between self-reported affect and observable emotional expression.

## Discussion

### Relationship between expressions and self-reported engagement

The results in this study demonstrated strong internal consistency across most measurement instruments, with the exception of the self-regulation component in cognitive engagement, which showed acceptable reliability. We observed significant correlations among behavioral and emotional (ρ = 0.876, p < 0.001) engagement, consistent with previous findings from traditional classroom research [5]. Furthermore, after reverse-coding negatively worded disaffection items to align their direction with engagement scales, we observed the anticipated correlations between engagement and disaffection dimensions (e.g., ρ = 0.724, p < 0.001 for behavior and ρ = 0.727, p < 0.001 for emotion dimension), consistent with established theoretical frameworks [62], validating the applicability of these scales in synchronous online learning environments.

Addressing our first research question, the results revealed that happiness expressions correlate significantly with several self-reported engagement measures, though not universally across all dimensions. Most importantly, we established a robust and significant correlation with emotional engagement (ρ = 0.31, p = 0.022), which represents our study’s primary validated finding and remained stable across our analytical approaches. This core relationship was further supported by correlations with specific emotional indicators: self-reported “happy” (ρ = 0.33, p = 0.015) and “loving” mood (ρ = 0.324, p = 0.017). These results align closely with the moderate correlation reported by Buono et al. (ρ = 0.38) using deep learning LSTM networks in a general online learning context [11], providing cross-validation for our findings. Furthermore, our results confirm that online second language learning, which incorporates grammar practice and speaking exercises, can similarly elicit learners’ emotional responses that manifest in detectable facial expressions [79,89].

Our multi-method validation approach provides preliminary support for using facial expressions as emotional engagement indicators in educational contexts. This approach combines facial expression recognition with self-reports, offering a potentially valuable complement to traditional assessment methods. While our factor analyses showed some consistency across different analytical approaches, we acknowledge important limitations in our measurement model, including the modest AVE value and small sample size. The emotional engagement factor showed different loading patterns, with emotional positivity displaying a larger difference (0.230) between EFA and CFA, likely due to prior information in Bayesian CFA. This integrated approach builds on existing technical foundations and may offer practical utility in educational contexts where emotional engagement is critical for learning success [2], though further validation with larger samples is needed to establish more robust psychometric properties.

This study also extended the previous studies focus in psychology and traditional face-to-face education that established connections between happiness expressions and emotional engagement [28,53,54,71], suggesting that facial expression recognition can serve as a valuable indicator of student engagement in synchronous online learning contexts [10,12]. From an educational perspective, our integration of facial expression recognition with established engagement metrics introduces a pioneering multi-method approach. This innovation addresses a critical gap in online learning research, where traditional engagement indicators—such as physical presence or verbal interaction—are often less observable [19,31,90]. As online education continues to grow, our methodology offers a scalable and objective framework for assessing engagement, with particular relevance to L2 contexts where emotional factors significantly influence learning processes [40].

The absence of significant correlations with behavioral engagement, cognitive engagement, and flow experience provides crucial boundary condition information that advances our theoretical understanding of what facial expression recognition can and cannot detect in educational contexts. Rather than representing methodological failures, these non-significant correlations should be interpreted as theoretically meaningful evidence about the specific scope and limitations of happiness expressions as engagement indicators.

These findings align with theoretical expectations about the distinct manifestation patterns of different engagement dimensions. Behavioral engagement primarily involves observable actions such as task completion, sustained attention, and active participation in interactions [22], which operate largely independently of internal emotional states. Students can demonstrate high behavioral engagement—completing tasks, maintaining focus, and participating actively—without necessarily experiencing or expressing happiness. Conversely, cognitive engagement represents internal mental processes including deep thinking, problem-solving, and strategic learning approaches [22,25] that may not consistently produce detectable facial changes. The complexity of flow as a psychological state, influenced by factors such as task difficulty, skill level, and individual differences [43], further explains why happiness expressions showed no significant correlation with flow experience.

Within Skinner’s framework, this pattern reflects the inherent complexity of emotional engagement itself. Emotional dissatisfaction encompasses both passive dissatisfaction (e.g., boredom) and active dissatisfaction (e.g., frustration, anger, sadness) [5,91]. While active positive emotions may manifest in detectable happiness expressions, passive states and negative emotions may not be captured through happiness indicators alone. The mood scale results support this interpretation, as only the “happy” and “loving” items demonstrated significant correlations with happiness expressions, while other emotional states showed no relationships.

This theoretical understanding is corroborated by our qualitative observations. Post-class interviews revealed multiple instances where participants reported high levels of enjoyment, focus, and engagement despite showing minimal happiness expressions during sessions. Some participants exhibited no detectable happiness expressions throughout entire teaching sessions yet reported positive learning experiences and cognitive engagement afterward. These findings demonstrate the asymmetric relationship between internal engagement states and external facial expressions: while positive engagement may sometimes manifest in detectable expressions, the absence of such expressions does not indicate disengagement, dissatisfaction, or lack of flow.

Additional complexity arises from contextual factors that can influence facial expressions independently of engagement levels. Previous studies have identified that learners may display happiness expressions due to non-learning factors such as time of day, external environmental changes [92,93], or individual personality differences [60]—factors that may be amplified in synchronous online teaching settings where students experience varying technical, environmental, and social conditions. This contextual variability underscores why happiness expressions, while valuable for assessing specific aspects of emotional engagement, have inherent limitations for detecting behavioral and cognitive dimensions.

Our findings reveal important limitations in binary engagement classification approaches. Unlike Zhang et al. and Siswantoro et al., who achieved high accuracy in binary engagement classification [10,13], our multidimensional analysis demonstrates that such simplified approaches may not capture the nuanced differences across engagement dimensions, particularly cognitive engagement and flow. This suggests significant limitations in relying solely on happiness expressions for comprehensive engagement assessment.

However, these limitations also point toward promising directions for future development. Savchenko et al. offer a potential solution by classifying multiple emotions alongside engagement in online learning [12], suggesting that future studies could incorporate a broader range of expressions (e.g., surprise, frustration, concentration indicators) to better capture complex emotional states and enhance the granularity of engagement detection. Our results support a multi-method approach where facial expression recognition serves as one component within comprehensive engagement assessment frameworks rather than a standalone solution.

Importantly, our findings suggest that the absence of happiness expressions should not be interpreted as indicating behavioral dissatisfaction, cognitive disengagement, or lack of flow state. Instead, these results position facial expression recognition as a targeted tool for assessing specific aspects of emotional engagement within broader, multi-method evaluation frameworks. This nuanced understanding helps establish realistic expectations for the technology while identifying its most appropriate applications in educational contexts.

In summary, while the correlation between expressions and emotional engagement has been widely established, differences in definitions and the complexity of emotional engagement in educational settings may lead to variations in implicitness. Overt expressions alone may not fully capture deeper states of emotional engagement, such as behavior engagement and flow [94]. Moreover, aligning with the findings of Goldberg et al. [69], the absence of a correlation with cognitive engagement, which was characterized by a higher level of implicitness [29], highlights the challenge of discerning implicit cognitive processes through facial expressions.

These findings contribute significantly to our understanding of engagement as a multifaceted construct and underscore the need for nuanced approaches to measurement and interpretation in educational research.

### Relationship between expressions and real-time reported engagement

Engagement is recognized as a dynamic phenomenon rather than a static one, and traditional single-time self-report scales often fall short in capturing this dynamic process [34,95]. The real-time engagement measurements employed in this study corroborate this understanding, although various methods of measuring engagement yield partially incongruent results.

Our findings build on prior work [15,67,71] by demonstrating that happiness expressions, when correlated with multiple engagement measures, provide a targeted indicator of emotional engagement in online L2 settings, extending the application of facial recognition beyond traditional classroom contexts. Specifically, the observed fluctuation in happiness expressions demonstrates notable similarities to the findings of Tonguç and Ozkara [68], indicating a moderate level of happiness at the onset of teaching sessions, followed by a decline and a subsequent resurgence reaching its peak at the conclusion. However, our results revealed a distinctive pattern: an earlier and more pronounced rebound in happiness expressions during the Grammar + Practice section, albeit for a shorter duration. This dynamic analysis, capturing engagement shifts across lesson phases, expands upon Buono et al.’s static correlations by offering a temporal perspective that enriches our understanding of engagement in L2 contexts [11]. This methodological advancement aligns with Siswantoro et al.’s CNN-based observations of real-time engagement modulation in synchronous virtual lectures and reinforcing the applicability of facial recognition in virtual classrooms [13].

This variation likely stems from factors that enhance participants’ display of happiness expressions, highlighting the fundamental malleability of engagement [2,34]. As Fredricks et al. [22] emphasized, learners demonstrate increased engagement under favorable interpersonal and environmental conditions, with teacher interventions and peer interactions serving as positive influences on student engagement [27,29]. The role of teachers becomes particularly crucial in online environments, where they must coordinate interactive processes and maintain engagement throughout the session [20]. While a direct comparison with Tonguç and Ozkara’s study [68] is limited by the lack of detailed instructional design information for specific time periods, our findings provide a foundation for future controlled experiments to validate engagement malleability.

Our comparative analysis of happiness expression recognition with observational data and students’ retrospective self-reports yielded nuanced insights that partially align with Goldberg et al.’s [69,96] findings. The observer reports confirmed students’ predominant passive engagement state, with statistically significant correlations between expressions and on-task active and passive classroom observations. However, our correlation coefficients (|ρ| < 0.306) were weaker than their reported significance (r = 0.45). This disparity likely stems from both methodological differences and the distinct context of their face-to-face classroom setting [69], where they integrated multiple indicators including facial expressions, head pose, and gaze. Therefore, the inclusion of multiple indicators may further enhance the accuracy of recognition. On the other hand, the correlations between happiness expressions and observed active disengagement (ρ = 0.059, p = .081) and passive disengagement (ρ = 0.035, p = .3) were very weak and non-significant. This further corroborates the conclusion drawn in the preceding section on single-time measurements: while happiness expressions may serve as cues for engagement, disengagement states tend to be associated with low facial activity and are difficult to detect through a single happiness expression metric alone [77].

The absence of correlation between classroom observations and students’ retrospective self-reports presents an interesting contrast with Goldberg et al.’s [69] findings while aligning with Skinner et al.’s [5] results. In Skinner’s study, the correlation between classroom observation results and both student and teacher reports were similarly weak. These may be attributed to observers or teachers potentially misinterpreting students’ behavior, construing compliant behavior as participation without a comprehensive understanding of learners’ emotions and cognition [91]. While active participation in synchronous classrooms – such as answering questions or engaging in activities – is readily observable, distinguishing between attentive silence and disengagement during passive periods remains challenging. Furthermore, the unbalanced attention paid to dissatisfaction emotions [97] compounds this issue, as teachers primarily focus on promoting active participation while potentially overlooking signs of passive dissatisfaction, particularly boredom.

Student self-reports are deemed more reliable than classroom observation [61]. In this study, the robust correlation between pleasure and focus (ρ = 0.882, p < 0.01) aligns with the findings of Grijpma et al. [96]. Their real-time interview investigation into changes in student participation suggested that once students engage or disengage in one-dimension, similar shifts may occur in other dimensions. Nevertheless, in this study, the recognition of happiness expressions also exhibited a significant correlation with students’ stimulus recall self-report results, albeit with a weak correlation coefficient.

## Conclusion and limitation

This study advances engagement research by introducing and validating a novel multi-method framework that systematically maps the specific capabilities and limitations of facial expression recognition in online L2 learning contexts. Our primary contribution lies in establishing facial expression recognition as a reliable indicator of emotional engagement while providing crucial boundary condition information about its limited effectiveness for detecting other engagement dimensions.

Our core empirical finding—a significant and stable correlation between happiness expressions and self-reported emotional engagement (ρ = 0.31, p = 0.022)—identifies emotional engagement as the dimension most accurately captured by facial expression analysis in synchronous online L2 classrooms. This finding remained robust across analytical approaches and aligns closely with previous research in online learning contexts [11], providing cross-validation for the utility of facial expressions in educational assessment. Complementary evidence from specific mood correlations (“happy” and “loving” items) further supports this targeted application.

Equally important are our systematic findings regarding what facial expression recognition cannot reliably detect. The absence of significant correlations with behavioral engagement, cognitive engagement, and flow experience provides theoretically meaningful evidence about the scope limitations of happiness expressions in educational settings. These boundary conditions reflect the asymmetric relationship between internal engagement states and observable expressions: while positive emotional engagement may manifest in detectable facial cues, behavioral and cognitive engagement operate through mechanisms that do not consistently produce observable happiness expressions.

Our temporal dual-perspective analysis further demonstrated that facial expression recognition successfully captures dynamic engagement fluctuations throughout learning sessions, showing modest but significant correlations with both retrospective self-reports and classroom observations. This temporal sensitivity represents a key advantage over traditional single-time assessment methods, enabling real-time monitoring of emotional engagement patterns.

These findings position facial expression recognition as a targeted tool for emotional engagement assessment within comprehensive, multi-method evaluation frameworks rather than as a standalone engagement detection solution. This nuanced understanding establishes realistic expectations for the technology while identifying its most appropriate and valuable applications in digital education contexts.

This study acknowledges several important limitations that inform the interpretation of our findings and directions for future research. First, our sample size (N = 54 for single-time measurements, N = 60 for real-time measurements), while adequate for detecting medium-to-large effects, was constrained by strict inclusion criteria and technical demands of facial expression recognition, but may have lacked sensitivity for smaller but potentially meaningful associations, particularly with cognitive engagement dimensions.

Second, our measurement model reveals important psychometric considerations for future research. The Average Variance Extracted (AVE = 0.459) below conventional thresholds indicates that facial expressions and self-reported emotional engagement capture complementary rather than identical aspects of emotional engagement. While this finding aligns with theoretical expectations that emotional engagement manifests across multiple, partially independent response modalities [74,75], future research with larger samples and diverse measurement approaches (e.g., physiological indicators, neurophysiological measures) is needed to establish more robust psychometric properties and determine whether this modest overlap represents a methodological limitation or a theoretically meaningful characteristic of comprehensive engagement assessment.

Third, our focus on happiness expressions alone may have constrained our detection capabilities. Students may exhibit engagement through other facial expressions (e.g., concentration indicators, surprise, or even mild frustration during challenging tasks) that were not systematically analyzed. Future studies incorporating broader expression recognition could enhance the granularity and scope of engagement detection.

Future research should prioritize several key directions to build upon our findings. Large-scale validation studies across diverse educational contexts and cultural settings are needed to establish the generalizability of our boundary condition findings. The integration of multi-modal indicators (gaze tracking, physiological measures, broader facial expression recognition) could address current limitations while maintaining the practical advantages of non-intrusive assessment. Additionally, longitudinal investigations could examine how the relationship between facial expressions and engagement evolves across different learning phases and individual development patterns.

Our findings emphasize that effective engagement assessment in digital learning environments requires multi-method approaches that leverage the specific strengths of different measurement modalities. Facial expression recognition offers valuable real-time insights into emotional engagement while complementing rather than replacing traditional assessment methods. This integrated approach represents a promising direction for enhancing both research precision and practical applications in online education.

## Supporting information

S1 Appendix Results of Spearman’s Rho Correlations Between Happiness Expression and Engagement Measurements. (DOCX)

S2 Appendix Results of Spearman’s Rho Correlations Between Happiness Expression and Mood Items. (XLSX)

S3 Appendix Results of single-time measurement. (XLSX)

S4 Appendix Results of real-time measurement. (XLSX)

S5 Appendix Complete Factor Analysis Results. (DOCX)
