# Supplementary material for: Facial expression recognition reveals students’ engagement in online class: Correlations with six engagement measurements
Source: PLoS One. 2025 Oct 22;20(10):e0334232. doi: 10.1371/journal.pone.0334232 (PMC12543194; doi:10.1371/journal.pone.0334232)
Supplement: S1 Appendix — (DOCX) [file pone.0334232.s001.docx]

**Appendix S1 Results of Spearman's Rho Correlations Between Happiness Expression and Engagement Measurements**

**TableS1 Correlations Between Happiness Expression and Single-time Measurements (*p*)**

| **Measurements** | **HappyExprAvg** | **BehavAvg** | **BehavPos** | **BehavNeg** | **EmoAvg** | **EmoPos** | **EmoNeg** | **CogAvg** | **StratUse** | **SlefReg** | **MoodScorded** |
| --- | --- | --- | --- | --- | --- | --- | --- | --- | --- | --- | --- |
| **BehavAvg** | 0.154 (0.265) | -- |  |  |  |  |  |  |  |  |  |
| **BehavPos** | 0.139 (0.318) | 0.888** (0.000) | -- |  |  |  |  |  |  |  |  |
| **BehavNeg** | 0.167 (0.227) | 0.944** (0.000) | 0.724** (0.000) | -- |  |  |  |  |  |  |  |
| **EmoAvg** | 0.152 (0.274) | 0.876** (0.000) | 0.762** (0.000) | 0.846** (0.000) | -- |  |  |  |  |  |  |
| **EmoPos** | 0.310* (0.022) | 0.785** (0.000) | 0.797** (0.000) | 0.711** (0.000) | 0.827** (0.000) | -- |  |  |  |  |  |
| **EmoNeg** | 0.117 (0.398) | 0.839** (0.000) | 0.706** (0.000) | 0.814** (0.000) | 0.979** (0.000) | 0.727** (0.000) | -- |  |  |  |  |
| **CogAvg** | 0.026 (0.850) | 0.408** (0.002) | 0.502** (0.000) | 0.311* (0.022) | 0.358** (0.008) | 0.526** (0.000) | 0.942** (0.000) | -- |  |  |  |
| **StratUse** | 0.112 (0.420) | 0.532** (0.000) | 0.629** (0.000) | 0.435** (0.001) | 0.423** (0.001) | 0.603** (0.000) | 0.270* (0.048) | 0.942** (0.000) | -- |  |  |
| **SlefReg** | 0.029 (0.834) | -0.014 (0.918) | -0.010 (0.943) | -0.011 (0.937) | 0.102 (0.463) | 0.247 (0.072) | 0.046 (0.744) | 0.615** (0.000) | 0.385** (0.004) | -- |  |
| **MoodScorded** | -0.030 (0.828) | 0.562** (0.000) | 0.608** (0.000) | 0.494** (0.000) | 0.497** (0.000) | 0.566** (0.000) | 0.454** (0.001) | 0.512** (0.000) | 0.532** (0.000) | 0.274* (0.045) | -- |
| **Flow** | 0.010 (0.941) | -0.126 (0.364) | -0.255 (0.062) | -0.085 (0.541) | 0.006 (0.965) | -0.034 (0.808) | 0.048 (0.728) | -0.169 (0.221) | -0.205 (0.136) | 0.051 (0.715) | 0.096  (0.491) |

| **TableS2 Correlations Between Happiness Expression and Real-time Measurements (*p*)** | | | | | | | | |
| --- | --- | --- | --- | --- | --- | --- | --- | --- |
| **Measurements** | **HappyExpr**  **Rel** | **On-Task** | **On-Act** | **On-Pas** | **Off-Task** | **Off-Act** | **Off-Pas** | **Pleasure** |
| **On-Task** | -0.047 (0.165) | -- |  |  |  |  |  |  |
| **On-Act** | 0.305** (0.000) | 0.035 (0.306) | -- |  |  |  |  |  |
| **On-Pas** | -0.306** (0.000) | 0.217** (0.000) | -0.961** (0.000) | -- |  |  |  |  |
| **Off-Task** | 0.065 (0.053) | -0.854** (0.000) | -0.028 (0.409) | -0.202** (0.000) | -- |  |  |  |
| **Off-Act** | 0.059 (0.081) | -0.557** (0.000) | 0.015 (0.652) | -0.121** (0.000) | 0.654** (0.000) | -- |  |  |
| **Off-Pas** | 0.035 (0.300) | -0.642** (0.000) | -0.050 (0.140) | -0.161** (0.000) | 0.751** (0.000) | -0.009 (0.787) | -- |  |
| **Pleasure** | 0.069* (0.042) | -0.019 (0.581) | -0.002 (0.960) | -0.004 (0.909) | -0.015 (0.653) | -0.008 (0.817) | -0.013 (0.696) | -- |
| **Arousal** | 0.106** (0.002) | -0.015 (0.668) | 0.032 (0.337) | -0.035 (0.307) | -0.025 (0.462) | -0.003 (0.925) | -0.030 (0.371) | 0.882** (0.000) |
| *. Correlation is significant at the 0.05 level (2-tailed).  **. Correlation is significant at the 0.01 level (2-tailed). | | | | | | | | |
