# Supplementary material for: Facial expression recognition reveals students’ engagement in online class: Correlations with six engagement measurements
Source: PLoS One. 2025 Oct 22;20(10):e0334232. doi: 10.1371/journal.pone.0334232 (PMC12543194; doi:10.1371/journal.pone.0334232)
Supplement: S5 Appendix — (DOCX) [file pone.0334232.s005.docx]

**Appendix S5: Complete Factor Analysis Results**

**A1. Exploratory Factor Analysis (EFA)**

**A1.1 Data Suitability Assessment**

Prior to conducting exploratory factor analysis, this study evaluated the suitability of the data. The Kaiser-Meyer-Olkin (KMO) test revealed an overall measure of sampling adequacy of 0.50, with individual MSA values of 0.50 for both variables. While this value falls within the marginal acceptable range, it supports the appropriateness of conducting factor analysis for exploratory purposes[1].

Bartlett's test of sphericity indicated that the correlation matrix significantly differed from an identity matrix (χ² = 5.97, df = 1, p = 0.015 < 0.05), satisfying the fundamental assumption for factor analysis and confirming that the data are suitable for factor analytic procedures.

**A1.2 Factor Number Determination**

This study employed both the Kaiser criterion and parallel analysis to determine the number of factors to extract[2, 3]. Eigenvalue analysis revealed that the first factor had an eigenvalue of 1.331, while the second factor had an eigenvalue of 0.669. The Kaiser-Guttman criterion supported extracting one factor (eigenvalue >1). Consistency across methods reinforces a single-factor structure. The consistency between these two methods supports the hypothesis of a single-factor structure.

**A1.3 EFA Results**

The results of the exploratory factor analysis based on a single-factor model are presented in Table S1. The analysis revealed that both observed variables loaded equally on the single factor with loadings of 0.575, indicating moderate factor loadings. The communality for each variable was 0.331, suggesting that approximately 33% of the variance in each variable is explained by the common factor.

**Table S6. Exploratory Factor Analysis Results (N = 54)**

| **Variable** | **Factor Loading** | **Communality (h²)** | **Uniqueness** |
| --- | --- | --- | --- |
| EmoPos | 0.575 | 0.331 | 0.669 |
| HappyExprAvg | 0.575 | 0.331 | 0.669 |

**Factor Statistics:**

- Sum of squared loadings: 0.661
- Proportion of variance explained: 33.08%
- Root mean square of residuals (RMSR): 0.000

Since a single-factor model with two variables is inherently saturated (df = -1), traditional fit indices are not applicable, as noted by Kline and Brown [4, 5]. The model achieves perfect fit by design due to having zero degrees of freedom, which is a fundamental characteristic of just-identified models in structural equation modeling.

**A2. Confirmatory Factor Analysis (CFA)**

**A2.1 Model Specification**

Based on the EFA results, this study constructed a single-factor confirmatory factor analysis model: positive_engagement =~ EmoPos + HappyExprAvg

**A2.2 Bayesian Prior Specification**

Considering the small sample characteristics and existing literature evidence, this study employed informative priors for Bayesian CFA analysis. Based on literature on self-report measures[6], the loading prior for EmoPos was set to normal (0.7, 0.15). Based on facial expression recognition research [7, 8], the loading prior for HappyExprAvg was set to normal (0.4, 0.1). Factor variance employed a gamma (1.2, 1) prior, while error variances used gamma (1.5, 1) priors.

**A2.3 CFA Results**

The results of the Bayesian confirmatory factor analysis are presented in Table S2. All parameters showed R̂hat values not exceeding 1.001, indicating excellent MCMC chain convergence. Standardized factor loadings revealed that EmoPos had a loading of 0.805 and HappyExprAvg had a loading of 0.572, both reaching statistical significance and conforming to theoretical expectations.

**Table S7. Bayesian Confirmatory Factor Analysis Results (N = 54)**

| **Parameter** | **Estimate** | **Post.SD** | **95% CI Lower** | **95% CI Upper** | **Std.lv** | **Std.all** | **R̂hat** |
| --- | --- | --- | --- | --- | --- | --- | --- |
| **Factor Loadings** |  |  |  |  |  |  |  |
| EmoPos | 1.000* | - | - | - | 0.529 | 0.805 | - |
| HappyExprAvg | 0.051 | 0.030 | 0.012 | 0.128 | 0.027 | 0.572 | 1.000 |
| **Error Variances** |  |  |  |  |  |  |  |
| EmoPos | 0.151 | 0.113 | 0.002 | 0.399 | 0.151 | 0.351 | 1.000 |
| HappyExprAvg | 0.002 | 0.001 | 0.000 | 0.003 | 0.002 | 0.673 | 1.000 |
| **Factor Variance** |  |  |  |  |  |  |  |
| positive_engagement | 0.280 | 0.101 | 0.116 | 0.494 | 1.000 | 1.000 | 1.000 |

*Fixed parameter for identification

**A2.4 Model Fit Evaluation**

Model fit assessment indicated a marginal log-likelihood of 20.259 and a posterior predictive p-value of 0.414, suggesting adequate model-data fit. Since this model is saturated, traditional chi-square tests are not applicable, but the posterior predictive check results support the model's adequacy.

**A2.5 Construct Validity Assessment**

**Table S8. Construct Validity Assessment**

| **Construct** | **Average Variance Extracted (AVE)** | **Composite Reliability (CR)** | **Discriminant Validity** |
| --- | --- | --- | --- |
| Positive Engagement | 0.459 | 0.629 | N/A (single factor) |

While the average variance extracted (AVE = 0.459) is marginally below the conventional threshold of 0.5, it is supported by significant factor loadings (> 0.57) and adequate composite reliability (CR = 0.629 > 0.60), which is consistent with guidelines for exploratory research using behavioral measures (Hair et al., 2019), indicating acceptable internal consistency for exploratory studies.

**A3. EFA-CFA Comparison and Validation**

**A3.1 Loading Consistency Analysis**

**Table S9. EFA vs CFA Loading Comparison**

| **Variable** | **EFA Loading** | **CFA Loading** | **Absolute Difference** |
| --- | --- | --- | --- |
| EmoPos | 0.575 | 0.805 | 0.230 |
| HappyExprAvg | 0.575 | 0.572 | 0.003 |

HappyExprAvg demonstrated excellent consistency across methods (difference = 0.003), while EmoPos showed moderate consistency. The loading difference for EmoPos can be attributed to the influence of prior information in Bayesian CFA. Overall, the loading pattern supports the robustness of the single-factor structure. Both analytical methods support the existence of a single "positive emotional engagement" factor. All factor loadings exceeded the practical significance threshold of 0.3 recommended by Hair et al. for exploratory research[9].

**A4. Sensitivity Analysis**

To evaluate the sensitivity of results to prior specifications, this study conducted sensitivity analysis using different prior settings (conservative, moderate, original). Results demonstrated some sensitivity of loading estimates to prior selection, which is typical in small-sample Bayesian analysis.

**Table S10. Prior Sensitivity Analysis Results**

| **Prior Setting** | **EmoPos Loading** | **HappyExprAvg Loading** | **Model Fit (PPP)** |
| --- | --- | --- | --- |
| Informed | 0.805 | 0.572 | 0.414 |
| Conservative | 0.430 | 0.918 | - |
| Moderate | 0.121 | 0.879 | - |

Despite variations in loading estimates, the main conclusions remain stable: both variables show significant loadings on the factor, supporting the hypothesized factor structure.

**A5. R Code for Replication**

*# ==========================================*

*# R Code for Factor Analysis of Facial Expression and Emotional Engagement*

*# Complete analysis workflow for academic replication*

*# ==========================================*

*# Load required packages*

packages <- c("blavaan", "lavaan", "psych", "haven", "tidyverse",

"corrplot", "effectsize", "ggplot2", "bayestestR")

lapply(packages, library, character.only = TRUE)

*# ==========================================*

*# 1. DATA PREPARATION*

*# ==========================================*

*# Load data (adjust file path as needed)*

raw_data <- haven::read_sav("your_data_file.sav")

*# Select analysis variables*

analysis_vars <- c("HappyExprAvg", "EmoPos", "EmoAvg", "HappyMood",

"BehavPos", "BehavAvg", "CogAvg", "MoodScorded")

*# Create clean dataset*

data_clean <- raw_data %>%

select(all_of(analysis_vars)) %>%

na.omit()

print(paste("Final sample size:", nrow(data_clean)))

*# ==========================================*

*# 2. DESCRIPTIVE STATISTICS*

*# ==========================================*

*# Generate descriptive statistics*

desc_stats <- psych::describe(data_clean)

print(round(desc_stats[, c("n", "mean", "sd", "min", "max", "skew", "kurtosis")], 3))

*# ==========================================*

*# 3. EXPLORATORY FACTOR ANALYSIS (EFA)*

*# ==========================================*

*# Prepare EFA data*

efa_vars <- c("EmoPos", "HappyExprAvg")

efa_data <- data_clean[, efa_vars]

cat("\n=== EXPLORATORY FACTOR ANALYSIS ===\n")

cat("Variables:", paste(efa_vars, collapse = ", "), "\n")

cat("Sample size:", nrow(efa_data), "\n")

*# Data suitability tests*

kmo_result <- psych::KMO(efa_data)

bartlett_result <- psych::cortest.bartlett(cor(efa_data), n = nrow(efa_data))

cat("\nKMO Test:\n")

print(kmo_result)

cat("\nBartlett's Test:\n")

print(bartlett_result)

*# Determine number of factors*

cat("\nParallel Analysis:\n")

parallel_result <- psych::fa.parallel(efa_data, fa = "fa", fm = "ml",

main = "Parallel Analysis Scree Plot")

eigenvalues <- eigen(cor(efa_data))$values

cat("Eigenvalues:", round(eigenvalues, 3), "\n")

cat("Recommended factors (Kaiser):", sum(eigenvalues > 1), "\n")

cat("Recommended factors (Parallel):", parallel_result$nfact, "\n")

*# Conduct EFA*

efa_1factor <- psych::fa(efa_data, nfactors = 1, rotate = "none", fm = "ml")

cat("\nEFA Results:\n")

print(efa_1factor, cut = 0.3, sort = TRUE)

*# Extract results*

loadings_matrix <- as.matrix(efa_1factor$loadings)

communalities <- efa_1factor$communality

variance_explained <- efa_1factor$values[1] / ncol(efa_data) * 100

cat("\nFactor Loadings:\n")

print(round(loadings_matrix, 3))

cat("\nCommunalities:\n")

for(i in 1:length(communalities)) {

cat(sprintf("%-15s: h² = %5.3f\n", names(communalities)[i], communalities[i]))

}

cat("\nVariance Explained:", round(variance_explained, 2), "%\n")

*# Create EFA summary table*

efa_summary_table <- data.frame(

Variable = rownames(loadings_matrix),

Factor_Loading = round(loadings_matrix[, 1], 3),

Communality = round(communalities, 3),

stringsAsFactors = FALSE

)

print(efa_summary_table)

*# ==========================================*

*# 4. CORRELATION ANALYSIS*

*# ==========================================*

cat("\n=== CORRELATION ANALYSIS ===\n")

*# Compute Spearman correlation matrix*

cor_matrix <- cor(data_clean, method = "spearman", use = "complete.obs")

print(round(cor_matrix, 3))

*# Visualize correlation matrix*

corrplot(cor_matrix,

method = "color",

type = "upper",

order = "hclust",

tl.cex = 0.8,

tl.col = "black",

title = "Spearman Correlation Matrix")

*# Test key relationship*

core_cor_test <- cor.test(data_clean$HappyExprAvg, data_clean$EmoPos,

method = "spearman")

print(core_cor_test)

*# ==========================================*

*# 5. BAYESIAN CONFIRMATORY FACTOR ANALYSIS (CFA)*

*# ==========================================*

cat("\n=== BAYESIAN CFA ANALYSIS ===\n")

*# Define measurement model*

bivariate_model <- '

*# Positive emotional engagement latent variable*

positive_engagement =~ EmoPos + HappyExprAvg

'

*# Set priors based on literature and EFA results*

informed_priors <- dpriors(

lambda = c("normal(0.7, 0.15)", *# EmoPos: self-report measure*

"normal(0.4, 0.1)"), *# HappyExprAvg: facial expression*

psi = "gamma(1.2, 1)", *# Factor variance*

theta = "gamma(1.5, 1)[sd]" *# Error variances*

)

cat("Running Bayesian CFA (this may take several minutes)...\n")

*# Fit Bayesian CFA*

bcfa_fit <- bcfa(

bivariate_model,

data = data_clean,

n.chains = 4,

burnin = 10000,

sample = 20000,

target = "stan",

dp = informed_priors,

bcontrol = list(

cores = 4,

control = list(adapt_delta = 0.99, max_treedepth = 15)

)

)

*# Display results*

cat("\nBayesian CFA Results:\n")

bcfa_summary <- summary(bcfa_fit, standardized = TRUE, fit.measures = TRUE)

print(bcfa_summary)

*# Extract standardized loadings*

standardized_loadings <- standardizedSolution(bcfa_fit)

factor_loadings <- standardized_loadings[standardized_loadings$op == "=~", ]

cat("\nStandardized Factor Loadings:\n")

for(i in 1:nrow(factor_loadings)) {

cat(sprintf("%-20s -> %-15s: λ = %5.3f [%5.3f, %5.3f]\n",

factor_loadings$lhs[i],

factor_loadings$rhs[i],

factor_loadings$est.std[i],

factor_loadings$ci.lower[i],

factor_loadings$ci.upper[i]))

}

*# ==========================================*

*# 6. SENSITIVITY ANALYSIS*

*# ==========================================*

cat("\n=== SENSITIVITY ANALYSIS ===\n")

*# Alternative prior specifications*

conservative_priors <- dpriors(

lambda = c("normal(0.5, 0.3)", *# More conservative priors*

"normal(0.3, 0.3)")

)

moderate_priors <- dpriors(

lambda = c("normal(0.7, 0.15)", *# Moderate priors*

"normal(0.5, 0.2)")

)

*# Fit alternative models*

bcfa_conservative <- bcfa(bivariate_model, data = data_clean,

dp = conservative_priors,

burnin = 5000, sample = 10000)

bcfa_moderate <- bcfa(bivariate_model, data = data_clean,

dp = moderate_priors,

burnin = 5000, sample = 10000)

*# Compare results across different priors*

loadings_original <- standardizedSolution(bcfa_fit)[1:2, "est.std"]

loadings_conservative <- standardizedSolution(bcfa_conservative)[1:2, "est.std"]

loadings_moderate <- standardizedSolution(bcfa_moderate)[1:2, "est.std"]

sensitivity_comparison <- data.frame(

Variable = c("EmoPos", "HappyExprAvg"),

Informed_Prior = round(loadings_original, 3),

Conservative_Prior = round(loadings_conservative, 3),

Moderate_Prior = round(loadings_moderate, 3)

)

cat("\nSensitivity Analysis - Loading Comparison:\n")

print(sensitivity_comparison)

*# ==========================================*

*# 7. REGRESSION ANALYSIS*

*# ==========================================*

cat("\n=== REGRESSION ANALYSIS ===\n")

*# Model 1: Simple regression*

model1 <- lm(EmoPos ~ HappyExprAvg, data = data_clean)

*# Model 2: Multiple regression with controls*

control_vars <- setdiff(names(data_clean), c("EmoPos", "HappyExprAvg"))

formula_str <- paste("EmoPos ~ HappyExprAvg +", paste(control_vars, collapse = " + "))

model2 <- lm(as.formula(formula_str), data = data_clean)

*# Display results*

cat("\nModel 1: Simple Regression\n")

print(summary(model1))

cat("\nModel 2: Multiple Regression with Controls\n")

print(summary(model2))

*# Effect sizes*

eta2_model1 <- eta_squared(model1)

eta2_model2 <- eta_squared(model2)

cat("\nEffect Size Analysis:\n")

cat("Model 1 (Simple) - eta² =", round(eta2_model1$Eta2[1], 4), "\n")

cat("Model 2 (Multiple) - HappyExprAvg eta² =", round(eta2_model2$Eta2[1], 4), "\n")

*# Model comparison*

anova_result <- anova(model1, model2)

cat("\nModel Comparison (ANOVA):\n")

print(anova_result)

**References**

1. Field A. Discovering statistics using IBM SPSS statistics. 5th ed: SAGE Publications; 2018 2018.

2. Kaiser HF. The application of electronic computers to factor analysis. Educational and psychological measurement. 1960;20(1):141–51. doi: 10.1177/001316446002000116.

3. Kaiser HF. An index of factorial simplicity. Psychometrika. 1974;39(1):31–6.

4. Brown TA. Confirmatory factor analysis for applied research. 2015.

5. Kline RB. Principles and practice of structural equation modeling. 2016.

6. Skinner EA, Kindermann TA, Furrer CJ. A motivational perspective on engagement and disaffection: Conceptualization and assessment of children's behavioral and emotional participation in academic activities in the classroom. Educational and psychological measurement. 2009;69(3):493–525. doi: 10.1177/0013164408323233.

7. Goldberg P, Sümer Ö, Stürmer K, Wagner W, Göllner R, Gerjets P, et al. Attentive or not? Toward a machine learning approach to assessing students’ visible engagement in classroom instruction. Educational Psychology Review. 2021;33:27–49. doi: 10.1007/s10648-019-09514-z.

8. Buono C, P. DC, B. DE, F. Assessing student engagement from facial behavior in on-line learning. Multimed Tools Appl. 2023;82:12859–77. doi: 10.1007/s11042-022-14048-8.

9. Hair JF, Risher JJ, Sarstedt M, Ringle CM. When to use and how to report the results of PLS-SEM. European business review. 2019;31(1):2–24. doi: 10.1108/EBR-11-2018-0203.
